# Global Epidemiology of Mucormycosis

**DOI:** 10.3390/jof5010026

**Published:** 2019-03-21

**Authors:** Hariprasath Prakash, Arunaloke Chakrabarti

**Affiliations:** Department of Medical Microbiology, Postgraduate Institute of Medical Education and Research, Chandigarh 160012, India; harisath2003@gmail.com

**Keywords:** mucormycosis, incidence, diabetes mellitus, haematological malignancy, *Rhizopus arrhizus*

## Abstract

Mucormycosis is an angio-invasive fungal infection, associated with high morbidity and mortality. A change in the epidemiology of mucormycosis has been observed in recent years with the rise in incidence, new causative agents and susceptible population. The rise has been perceived globally, but it is very high in the Asian continent. Though diabetes mellitus overshadow all other risk factors in Asia, post-tuberculosis and chronic renal failure have emerged as new risk groups. The rhino-cerebral form of mucormycosis is most commonly seen in patients with diabetes mellitus, whereas, pulmonary mucormycosis in patients with haematological malignancy and transplant recipients. In immunocompetent hosts, cutaneous mucormycosis is commonly seen following trauma. The intriguing clinical entity, isolated renal mucormycosis in immunocompetent patients is only reported from China and India. A new clinical entity, indolent mucormycosis in nasal sinuses, is recently recognized. The causative agents of mucormycosis vary across different geographic locations. Though *Rhizopus* *arrhizus* is the most common agent isolated worldwide, *Apophysomyces* *variabilis* is predominant in Asia and *Lichtheimia* species in Europe. The new causative agents, *Rhizopus homothallicus*, *Mucor irregularis*, and *Thamnostylum lucknowense* are reported from Asia. In conclusion, with the change in epidemiology of mucormycosis country-wise studies are warranted to estimate disease burden in different risk groups, analyse the clinical disease pattern and identify the new etiological agents.

## 1. Introduction

Mucormycosis is caused by the fungi belonging to the order *Mucorales*. Humans acquire the infection predominantly by inhalation of sporangiospores, occasionally by ingestion of contaminated food or traumatic inoculation [1,2]. The fungi under *Mucorales* are ubiquitous, and morphologically appear as broad, aseptate or sparsely septate ribbon-like hyphae. Eleven genera and ~27 species under *Mucorales* are associated with human infections. *Rhizopus arrhizus* is the most common agent causing mucormycosis across the globe, followed by *Lichtheimia*, *Apophysomyces*, *Rhizomucor*, *Mucor* and *Cunninghamella* species [3,4,5].

Mucormycosis is associated with angio-invasion and high mortality [4,5]. The infection is increasingly reported in patients with diabetes mellitus, haematological malignancy, solid organ transplants, and corticosteroid therapy [5,6,7,8,9]. A difference in the prevalence of risk factors/underlying disease and causative agents of mucormycosis have been reported between the western world and Asian countries [4]. Diabetes mellitus is the most common risk factor in the Asian continent, whereas haematological malignancies and transplantation are the major risk factors in European countries and the United States [10,11]. A considerable number of cases are documented following trauma in apparently healthy individuals. In recent years, health-care-associated mucormycosis is increasingly documented [12]. The present review discusses the global incidence of mucormycosis with the change in the epidemiological features including causative agents, risk factors or underlying disease.

## 2. Incidence of Mucormycosis

The incidence of mucormycosis is rising globally [6,7,8,9,13,14,15], but the rise is very high in India and China among patients with uncontrolled diabetes mellitus [5,10,16,17,18]. However, a recent review of 851 cases over the period January 2000 through January 2017, provides a different indication that the disease burden is higher in Europe than in Asia, as they reported 34% in Europe, followed by Asia (31%) and North or South America (28%), Africa (3%), Australia and New Zealand (3%) [4]. The contrary data may be due to under-reporting during this period from Asian countries. In reality, a rising number of cases are reported from India [5,10,16,17].

### 2.1. Population or Hospital-Based Estimates

The population-based prevalence of mucormycosis is reported from a few countries. In San Francisco, California, a population-based surveillance study conducted during 1992 to 1993 documented the cumulative annual prevalence of invasive fungal infections at 178.3 per million, with mucormycosis at 1.73 cases per million [6]. An autopsy-based study from a tertiary care cancer centre in the United States documented a rise in the incidence of mucormycosis in haematological malignancy patients from 0.006 cases per 100 autopsies in 1989–1993 to 0.018 cases in 2004–2008 [19]. The prevalence of mucormycosis related hospitalizations was estimated at 0.12 per 10,000 discharges by a retrospective case analysis of 560 hospitals with 104 million patients in the United States from January 2005 to June 2014 [14]. The rise in the prevalence of mucormycosis had been reported in few reports from Europe. A multicentre study from Spain reported the prevalence of mucormycosis at 0.43 cases per million population/year and 0.62 cases/100,000 hospital admissions [7]. In another single centre study from Spain documented the rise in the prevalence of mucormycosis from 1.2 cases/100,000 admissions (1988 to 2006) to 3.3 cases/100,000 admissions (2007 to 2015) [15]. In France, a rise of mucormycosis cases was reported based on nation-wide population-based study, with prevalence rate of 0.7 cases per million in 1997 rose to 1.2 per million in 2006 [8]. From Switzerland, a rise in prevalence from 0.57 cases/100,000 admissions/year (before 2003) to 6.3 cases/100,000/ admissions/ year (after 2003) was noted at a centre, and the rise was presumed to be due to the increased use of voriconazole and caspofungin prophylaxis [9]. From Belgium, Saegeman et al. reported a rise from 0.019 cases/10,000 patient-days during 2000 to 0.148 cases/10,000 patient-days during 2009 [13]. A similar rise in prevalence had been reported in a few studies from Asia. A recent study from India reported a rise of mucormycosis cases from 24.7 cases per year (1990–2007) to 89 cases per year (2013–2015) at a single tertiary-care hospital [5]. In a multicentre study from Indian ICUs, mucormycosis has been reported at 24% of all invasive mould infections [20]. Dolatabadi et al. from Iran reported a rising trend of mucormycosis from 9.7% in 2008 to 23.7% in 2014 [21]. A national survey on medical autopsies at Japan reported that the rate of mucormycosis increased from 0.01% cases in 1969 to 0.16% of cases in 1989 [22].

### 2.2. Estimated Incidence

The true incidence/prevalence may be more in mucormycosis, as many of the cases remain undiagnosed due to difficulty in collecting the sample from deep tissue and low sensitivity of diagnostic tests. The Leading International Fungal Education (LIFE) portal has estimated the burden of serious fungal infections globally. According to their estimate, the annual prevalence of mucormycosis might be around 10,000 cases in the world barring India. After the inclusion of Indian data, the estimate of mucormycosis rose to 910,000 cases globally [23,24]. The estimated burden of mucormycosis in different countries is summarised in Table 1 [25,26,27,28,29,30,31,32,33,34,35,36,37,38,39,40,41,42,43,44,45,46,47,48,49,50,51,52,53,54,55,56,57,58,59,60,61,62,63]. The estimated incidences per million populations in different continents were: Europe (from 0.2 cases in Denmark to 95 cases in Portugal), USA (3.0 cases), Canada (1.2 cases) and Australia (0.6 cases). A computational-based approach estimated the prevalence of mucormycosis at 140 cases per million populations in India, with the prevalence ranging between 137,807 cases to 208,177 with the mean of 171,504 (SD: 12,365.6; 95% CI: 195,777–147,688) and a mean attributable mortality at 65,500 (38.2%) per year [24].

## 3. Underlying Disease/Predisposing Factors

**Figure 1:**
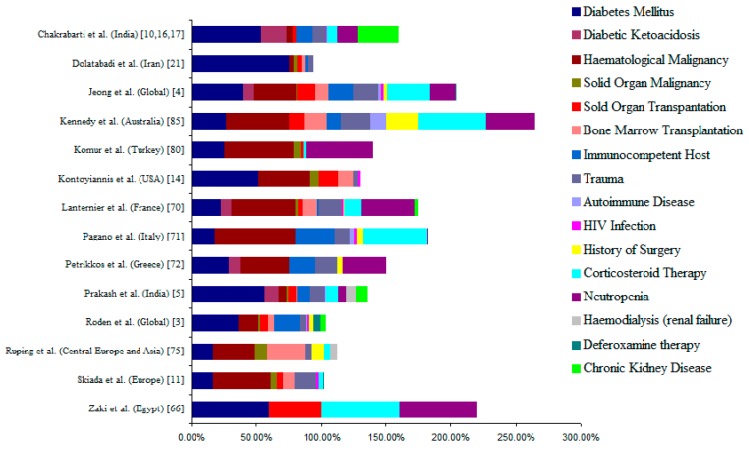
Underlying disease/ risk factors associated with mucormycosis. The percentage of values given in the figure was calculated based on the data given in the literature. Percentage counts in total are more than actual values, because multiple risk factors are counted more than once. The data was pooled from studies by Chakrabarti et al. 2006, 2001, 2009 [10,16,17].

The common underlying diseases in mucormycosis include diabetes mellitus with or without diabetic ketoacidosis, malignancies (haematological and solid organ tumor), transplant recipients (haematopoietic stem cell and solid organ transplants), corticosteroid therapy and neutropenia. However a considerable number of cases have been reported in patients without any underlying disease or risk factors. Figure 1 depicts the major risk factors/underlying disease documented in different studies across the globe.

A contrasting difference in underlying disease/risk factors has been noted between the western world (USA and Europe) and the Asian region. Diabetes mellitus is the most common risk factor associated with mucormycosis in India [10,16,17], contrasting to haematological malignancy and transplant recipients in Europe and the United States [11,14]. Though a rise in the number of cases with haematological malignancies and transplantation as risk factors in India has been noted in recent years, huge number of cases with diabetes overshadows the picture [64]. 

Diabetes mellitus as predisposing factor varies from 17% to 88% globally [11,65,66]. Three major case series from India reported diabetes as a risk factor over 50% cases with mucormycosis [10,16,17], compared to 36–40% of global data in two separate meta-analysis studies [3,4]. A recent multi-centre study from India documented that 57% of their patients had uncontrolled diabetes mellitus with 10% diabetic ketoacidosis, and diabetes as a risk factor was significantly higher in north Indian centres compared to south India [5]. Another study from the same country reported 35 cases of mucormycosis in 22,316 consecutive diabetic patients indicating the prevalence of mucormycosis at 1.6 cases per 1000 diabetics [67]. The majority of the population in India does not have regular health check-up and mucormycosis was a diabetes-defining illness in 23% to 43% patients as reported in multiple publications [10,17]. Compliance to anti-diabetic therapy is also poor in India and a large number of diabetics remain uncontrolled. It is claimed that regular statin use in diabetic patients has brought down diabetes as a risk factor for mucormycosis in the United States [68], though a case series from the same country reported diabetes as risk factor in 52% cases of mucormycosis [14]. Diabetes has also been reported as an important risk factor in Iran (74%) and Mexico (72%) [20,69]. In comparison to other regions, this risk factor is lower in European studies ranged from 17% to 29%: 17% in European Confederation of Medical Mycology (ECMM) study [11]; 23% in RetroZygo Study of France [70], 18% in Italy [71], 29% in Greece [72].

The prevalence of diabetic population is estimated to rise from 366 million in 2011 to 522 million in 2030 globally [73]. China topped the list with an estimated rise from 90 million in 2011 to 129.7 million in 2030, followed by India (61.3 million in 2011 to 101.2 million in 2030) and the United States (23.7 million in 2011 to 29.6 million in 2030). Similarly, the rise has been postulated in Brazil, Japan, Mexico, Egypt, and Indonesia. Considering the rise in diabetic population, the rise in the number of mucormycosis cases is also predicted (Table 1).

Haematological malignancy (HM) was the most common underlying disease for mucormycosis in Europe and the United States, ranging from 38% to 62% (Figure 1). Patients with acute myeloid leukaemia, myelodysplastic syndrome, haematopoietic stem cell transplant (HSCT) and acute lymphoblastic leukaemia are at greater risk of acquiring mucormycosis during the neutropenic phase [3,74,75]. A multicentre cohort study from France reported mucormycosis cases in 0.4% allogeneic HSCT recipients [76]. A retrospective cohort study in HSCT patients from Italy reported <0.1% incidence of mucormycosis during 1999–2003 [77]. In the United States, TRANSNET study on HSCT patients reported mucormycosis comprised 8% of invasive fungal infections, with an annual cumulative prevalence at 0.29% [78,79]. HM was reported as a risk factor in 3.4% mucormycosis cases in Iran, 6.3% in India and 13.6% in Mexico [5,21,69]. 

Solid organ malignancies and solid organ transplant (SOT) recipients are also important risk factors for mucormycosis though in a small proportion of cases (Figure 1). Various studies reported SOT as an underlying disease in 2–15% mucormycosis cases [4,5,14,80]. TRANSNET study reported the overall 12-month cumulative prevalence of mucormycosis at 0.07% in SOT patients [78,81]. Almyroudis et al. reported two mucormycosis cases per 1000 SOT recipients [82]. However, the incidence of mucormycosis may vary depending on SOT types: in renal recipients (0.4–0.5 cases per 1000 patients), in liver recipients (4–16 per 1000 patients), in heart recipients (8 per 1000 patients) and in lung recipients (13.7–14.0 per 1000 patients) [82]. Among SOT recipients, the patients with renal failure (58%), diabetes mellitus (38%) and prior antifungal prophylaxis with voriconazole or caspofungin (26%) are more prone to develop mucormycosis [83]. Immunosuppressive therapy and high steroid dose (≥600 mg of prednisone) in those patients make the patient susceptible to mucormycosis [82]. Corticosteroid impairs the macrophage and neutrophil function and makes the patient susceptible [84]. Apart from SOT recipients, steroids are often used in the treatment of autoimmune disorders. Kennedy et al. reported an autoimmune disease as the underlying disease in 12% mucormycosis cases [85]. A global review documented 2% of the patients with mucormycosis had an autoimmune disease as underlying illness [4].

Iron overload and deferoxamine therapy play a major role in the pathogenesis of mucormycosis. Earlier deferoxamine was commonly used to reduce iron/aluminium overload in patients with diabetic ketoacidosis (DKA), haemodialysis, renal failure and transfusion related disorders [86]. The iron removed by deferoxamine is captured by siderophores on *Rhizopus* species and iron helps in the growth of those fungi. An international registry on dialysis patients registered 59 cases of mucormycosis and 70% of those patients had no known underlying disease. However, 78% of the patients had deferoxamine therapy, which made the authors suspicious about deferoxamine therapy as a risk factor for mucormycosis [87]. The mortality was very high (80%) in those patients who were on deferoxamine therapy and developed mucormycosis [3,87]. However, the new iron chelators like deferasirox and deferiprone effectively chelate the iron without predisposing the patients to mucormycosis [88,89]. An open-labelled deferasirox therapy in eight proven cases of mucormycosis patients showed no adverse effects [88]. However, the same group of authors conducted a randomized, double-blinded, placebo-controlled trial to determine the efficacy and safety nature of deferasirox and liposomal Amphotericin B therapy (DEFEAT Mucor study) for mucormycosis. The mortality rate was higher in liposomal amphotericin B plus deferasirox treated group (82%) in comparison to only liposomal amphotericin B treated group (22%). Thus, the study did not support the use of deferasirox as an adjuvant therapy in the treatment of mucormycosis, though patients under deferasirox treated arm had higher morbidity [90]. A recent retrospective study on deferiprone as adjunctive therapy showed better safety and tolerability with a successful outcome in 67% of the treated patients [89].

Lionakis et al. reviewed breakthrough invasive mould infection after antifungal prophylaxis in haematological malignancy and HSCT patients [91]. The study reported breakthrough mucormycosis after prophylaxis with azoles and echinocandins. Similarly, a recent meta-analysis documented 92 cases of mucormycosis in patients with prior antifungal prophylaxis; voriconazole (52%), fluconazole (25%), itraconazole (7.6%), posaconazole (5%) and caspofungin (9.8%) [4]. Breakthrough mucormycosis was first reported after voriconazole therapy. Marty et al., 2016 reported an increased frequency of mucormycosis cases after treatment with voriconazole prophylaxis in HSCT patients [92]. Similarly, multiple studies documented breakthrough mucormycosis after voriconazole prophylaxis [93,94,95,96]. However, a multi-centre randomized double blind clinical trial in HSCT recipients reported no significant difference in mucormycosis cases between the patients treated with voriconazole or fluconazole prophylaxis [97]. Posaconazole was commonly used as salvage therapy for mucormycosis. However, Auberger et al., 2012 reported 55% of proven mucormycosis cases after posaconazole prophylaxis [98]. Similarly, other studies reported 15 to 22% of mucormycosis cases after treatment with posaconazole [99,100]. Recently, isavuconazole was approved by the FDA for the treatment of mucormycosis; various studies reported 17 to 30.8% cases of breakthrough mucormycosis even after isavuconazole prophylaxis [101,102,103]. Similarly, breakthrough mucormycosis has been reported after echinocandin prophylaxis in 10-14% of mucormycosis cases [104,105,106]. A recent study reported six cases of breakthrough mucormycosis after micafungin prophylaxis [107]. A stringent case control study is essential to investigate the association of antifungal agents in breakthrough mucormycosis and clinicians should be aware of the risk of mucormycosis in high risk populations with prior antifungal prophylaxis.

Other risk factors associated with mucormycosis are HIV infection, intravenous drug use, low birth weight infants, malnutrition, chronic alcoholism, liver diseases, chemotherapy and use of calcineurin inhibitors [4,5]. A recent study from India reported chronic kidney disease (8.9%) and post-pulmonary mucormycosis (6.9%) as emerging risk factors of mucormycosis [5]. Moreira et al., 2016 reviewed 67 cases of mucormycosis in HIV patients and reported intravenous drug use (50%) as a major predisposing factor, followed by neutropenia (29.7%), corticosteroid use (15%) and diabetes mellitus (15.6%). Those patients generally present with disseminated form of mucormycosis [108]. Though Jeong et al., 2019 reported 16% of their patients with mucormycosis were on calcineurin inhibitors, other study recorded reduced risk of mucormycosis after tacrolimus, a calcineurin inhibitor therapy in SOT recipients [4,83]. Calcineurin inhibitors are found to act synergistically with other antifungal agents and improve antifungal efficacy and better survival of the host [109,110,111].

Mucormycosis is not only the disease of the immunocompromised host; a considerable number of cases are seen in an immunocompetent host without any known underlying illness. Two meta-analysis at different time periods documented ~19% of the mucormycosis cases occur in immunocompetent hosts [3,4]. Those patients often present with cutaneous mucormycosis after trauma, burns, surgery, use of contaminated dressings and injection [3,4]. In China and India, a distinct clinical entity of isolated renal mucormycosis was observed in apparently healthy individuals [10,17,112]. 

## 4. Healthcare Associated Mucormycosis

Though mucormycosis was considered a community-acquired disease, nosocomial mucormycosis has been increasingly reported from many hospitals [12,113]. Documented cases of mucormycosis have been noted after use of the contaminated umbilical catheter and elastoplast adhesive dressings [114]. Outbreaks associated with contaminated wooden tongue depressors [115,116], wooden sticks [117], karaya ostomy bags [118] and bandages [119] have been described. The underlying diseases of those patients included diabetes mellitus (22%), solid organ transplantation (24%), steroid therapy (37%), and malignancy (12%). The skin was the most common site involved (57%), followed by gastrointestinal tract (15%), lungs (8%), sinuses and brain (4%). Disseminated infection was reported in 2% of patients [12]. An outbreak of intestinal mucormycosis had been reported in a haematology ward of a hospital over six months period in China possibly after ingestion of allopurinol tablets and ready-to-eat food. Corn-starch was possibly the source of contamination, as it was used to prepare both preparations [120]. Duffy et al. reported outbreak associated with contaminated linens in the United States hospital and *Rhizopus* species was isolated from 42% of the samples collected from clean linens [121]. Since invasive mucormycosis is found to be an important cause of mortality in debilitated patients, a high index of suspicion should exist among the clinicians to predict the outbreaks in the hospital environment.

## 5. Clinical Forms of Mucormycosis

**Figure 2:**
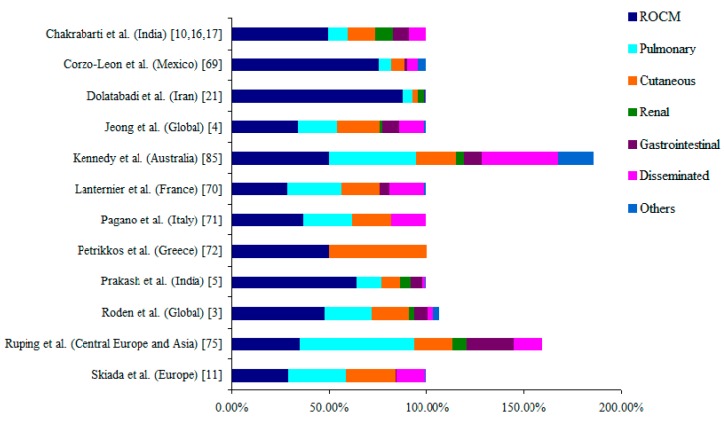
Clinical forms of mucormycosis reported from different studies across the globe. The percentage of values given in the figure was calculated based on the data provided in the literature. Multiple sites of infection were reported in few studies: Roden et al. 2005 [3], Ruping et al. 2010 [75], Kennedy et al. 2016 [85]. The data was pooled from studies by Chakrabarti et al. 2006, 2001, 2009 [10,16,17].

**Figure 3:**
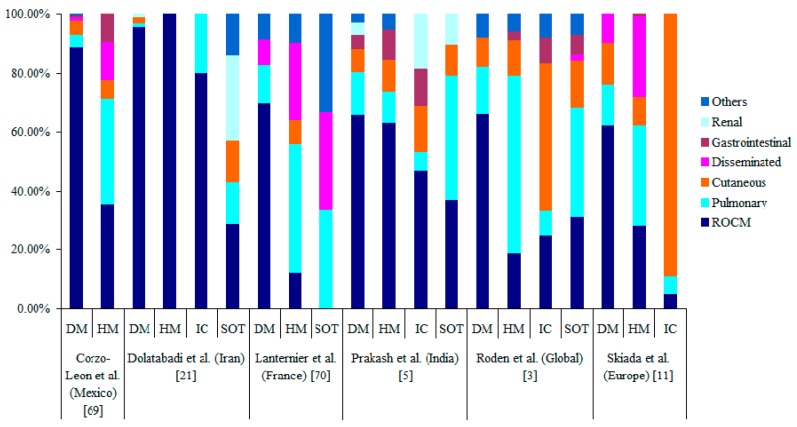
Clinical forms of mucormycosis in various underlying disease. Abbreviations: DM: Diabetes Mellitus, HM: Haematological Malignancy, IC: Immunocompetent and SOT- Solid Organ Transplant.

Based on the anatomical site of involvement, mucormycosis is classified into rhino-orbito-cerebral (ROCM), pulmonary, gastrointestinal, cutaneous, renal, disseminated and other miscellaneous forms, which include infection of bones, heart, ear, parotid gland, uterus, urinary bladder and lymph nodes [3,4]. The clinical forms of mucormycosis reported in different studies are shown in Figure 2. Multiple studies reported the specific association of underlying disease/predisposing factors with clinical types of mucormycosis. Figure 3 depicts the different clinical types of mucormycosis in various underlying disease.

### 5.1. Rhino-Orbito-Cerebral Mucormycosis (ROCM)

ROCM is the most common form and it is often seen in patients with diabetic ketoacidosis or with uncontrolled diabetes mellitus [3,4]. A study from India reported that 88% of the patients with ROCM had diabetes mellitus [65]. A similar finding was reported from the United States, where 83% of the patient had diabetes mellitus [122]. Diabetes was a risk factor in 51% to 64% of the ROCM cases in other studies [4,123,124]. Solid organ transplant, corticosteroid therapy, chronic kidney disease and intravenous drug use were also found as risk factors in ROCM cases [3,4,5]. Cerebral extension with high mortality was noted in SOT recipients [125]. The common non-ophthalmic symptoms include fever (26–44%), headache (5–25%), facial swelling (27–34%), facial pain (3–22%), nasal discharge (2–18%), epistaxis (0.5–9%), sinusitis (1–26%), hemiplegia (0–4%), nasal ulceration (3–38%), palatal eschar (5–14%), tooth ache (0.5–3.5%), facial numbness (3–7%), facial nerve palsy (0.5–11%), bone destruction and altered mental status (2–22%). Orbit is involved by direct extension of the disease from the paranasal sinuses. The ophthalmic signs and symptoms include eye pain, decreased vision, ophthalmoplegia (15–29%), proptosis (11–16%, chemosis (4.5–9%), ptosis (3.5–18%), orbital cellulitis (2–16%), periorbital discolouration and necrosis (2–4%) [123,124]. Infection can extend to the brain from the sino-nasal area or from retro-orbital region by extending through ethmoid and sphenoid sinuses or from superior orbital fissure by perineural route or through cribiform plate [126]. Occasionally, cerebral mucormycosis can happen through haematogenous route from distinct organs.

The imaging studies may help to delineate the extent of tissue invasion. The computed tomography and magnetic resonance of paranasal sinus mucormycosis included mucosal thickening, osseous erosion, sinusitis with hypo/mild/hyper intense lesions, and destruction of bones in nasal septa, orbit, maxilla and mandible. In case of orbital and cerebral extension, the disease can be seen as orbital cellulitis, optic neuritis, soft tissue infiltration in the optical apex, bone rarefaction and erosion of the skull base, cavernous sinus and internal carotid artery thrombosis, infracts and intra-cranial abscess in the brain [127,128].

### 5.2. Pulmonary Mucormycosis

Pulmonary type is the second most common site of involvement and often seen in patients with haematological disorders and transplant recipients [4,5]. Haematological malignancy was the major risk factor (32–40%), followed by diabetes mellitus (32–56%), haematopoietic stem cell transplant (1–9.8%) and solid organ transplant (6.5–9%) and renal disease (13–18%) in pulmonary mucormycosis [129,130,131]. Prakash et al. reported post-pulmonary tuberculosis (21%) as one of the risk factors for pulmonary mucormycosis [5]. The patients often present with high fever (38–70%), persistent cough (50–61%), pleuritic chest pain (22–37%), dyspnoea (19–34%), and haemoptysis (16–28%). The diagnosis of pulmonary mucormycosis remains a challenge. Imaging studies can be non-specific. The patients may present with lung infiltration and consolidation (58–96%), multiple nodules, pleural effusion (6–21%), thickly walled cavities (6–37%), hilar or mediastinal lymphadenopathy (3.3%), air crescent sign (1.1–8%) and pneumothorax (1–3%) on imaging studies. The reverse halo sign, the characteristic sign of mucormycosis, was seen only in 9.8% of the patients [129,130,131]. Pulmonary mucormycosis is usually unilateral (62–75%), occasionally bilateral (16–25%), rarely hilar or mediastinal (3%). In unilateral lung disease, upper lobe is commonly involved (40–45%), followed by lower lobe (16–21%) and middle lobe (1–3%); the multi-lobar disease is seen in 6–12% of the pulmonary mucormycosis cases [130,131].

### 5.3. Cutaneous Mucormycosis

Cutaneous mucormycosis often occurs after trauma or breach of skin and can be seen in the immunocompetent host. A review on cutaneous mucormycosis reported that 43–67% of the patients were apparently immunocompetent [132]. Diabetes mellitus (10–15%) and SOT recipients (5–16%) may occasionally acquire cutaneous mucormycosis [132]. The major predisposing factor in cutaneous mucormycosis is penetrating trauma (23–88%). Other risk factors include intramuscular injection in sub-optimal healthcare facility (42%), open wound trauma (21%), motor vehicle accident (3–33%), surgery (8–30%), contaminated dressings (8–15%), burns (5–11%), natural disasters (5%), animal bites and scratches (9%) [3,4,10,132,133,134]. Based on the extent of the invasion, cutaneous mucormycosis can be classified as localized infection, deep extension, and as part of the disseminated infection. The localized infection is seen in 32–56% of the patients, usually restricted to the cutaneous and subcutaneous tissue without invading adjacent sites. Deep extension refers to the invasion of muscles, bones and tendons and it is noted in 24–52% of the patients. In these patients, the infection often presents as necrotising fasciitis with erythematous necrotic eschar. Cutaneous mucormycosis as a part of the disseminated infection refers to an infection involving other non-contiguous site besides cutaneous site and is seen in 16–20% of cutaneous infection [3,131,133]. 

### 5.4. Gastrointestinal Mucormycosis

Gastrointestinal (GI) mucormycosis is the most difficult disease to diagnose ante-mortem, and commonly seen in low birth weight infants, in patients with malnutrition or undergoing peritoneal dialysis [3,135]. Among classical immunocompromised hosts with gastrointestinal mucormycosis, the disease is commonly seen in patients with solid organ transplants (52%), haematological malignancies (35%) and neutropenia (38%) [135]. Whereas, in the non-classical groups of patients, diabetes mellitus (12.2%), chronic alcoholism (6.3%), malnutrition (16.7%), peritoneal dialysis (8.5%), and use of broad spectrum antibiotics (37.1%) are the major risk factors [135]. Diabetes mellitus and peritoneal dialysis are the major risk factors in the adult population, where as, broad spectrum antibiotics use and malnutrition are significantly associated with children. The most common site of infection is bowel (64.2%), which includes large intestine (43.2%), stomach (33%), small intestine (28.4%), and oesophagus (3.4%) [135,136]. The patients usually present with abdominal pain (35.3–68%), gastrointestinal bleed (34–48%), abdominal distension (49.7%) and diarrhoea (8%). 

### 5.5. Renal Mucormycosis

Various studies from India documented the rise in patients with isolated renal mucormycosis from 5.4% to 14% among all mucormycosis cases [5,10,16,17]. In China and India, 33–100% of isolated renal mucormycosis cases had no underlying illness [5,10,16,17,137]. Patients usually present with fever, flank pain, haematuria or anuria [17,138]. Computer tomography and ultrasound are helpful in the early diagnosis of renal mucormycosis. CT of the abdomen shows bilaterally enlarged kidneys with thickening of the renal pelvis, infarction in the parenchyma [138]. 

### 5.6. Disseminated Mucormycosis

As *Mucorales* are angio-invasive, and can disseminate through haematogenous route. A meta-analysis reported 13% of the mucormycosis cases present with disseminated disease. Lung (91.2%) is the most common site of dissemination followed by the central nervous system (53%), sinus (32.4%), liver (17.6%) and kidney (14.7%) [11]. Solid organ transplant recipients and haematological malignancy patients are at increased risk of disseminated mucormycosis [4]. 

## 6. Causative Agents of Mucormycosis

**Figure 4:**
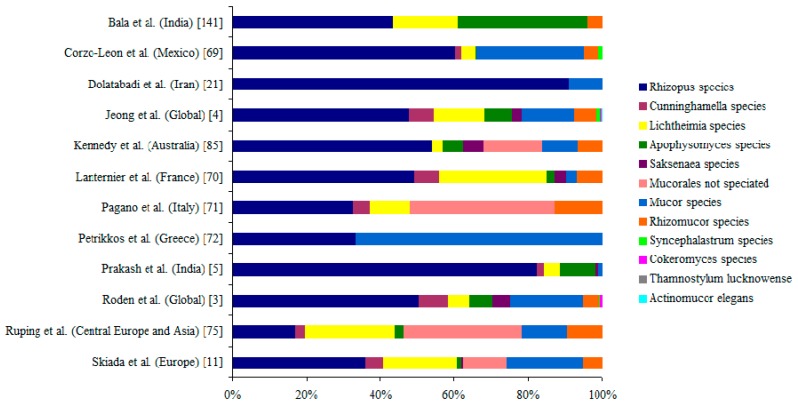
Causative agents of mucormycosis.

Eleven genus and ~27 species under the order *Mucorales* are known to cause mucormycosis [3,4,139]. Though microscopic morphology helps in presumptive identification, molecular techniques help in the accurate identification of the causative agents either from cultures or from tissue specimens. Figure 4 illustrates the species of *Mucorales* in various studies across the globe. Species under Genera *Rhizopus, Lichtheimia*, and *Mucor* are common causative agents and *Rhizopus arrhizus* is the most common agent [4]. A meta-analysis of mucormycosis cases revealed that *Rhizopus* species was often associated with ROCM form of the disease; *Cunninghamella* species with pulmonary disease or disseminated disease. Whereas, *Apophysomyces* and *Saksenaea* species were commonly isolated from cutaneous mucormycosis [4]. The ketoacidosis predisposes *Rhizopus* species and not *Lichtheimia*, where as corticosteroid predisposes *Lichtheimia* species [70]. Mortality associated with *Cunninghamella* species was found be significantly higher than infection with any other species under *Mucorales* [3,4]. A variation of species distribution was observed between different geographic regions. Infections due to *Lichtheimia* species are common in Europe, it is relatively rare in other regions [5,11,70]. *Apophysomyces variabilis* is the second most common cause of mucormycosis in India [4,140]. India accounts for approximately 60% of the documented mucormycosis cases due to *Apophysomyces* species [5,140,141]. Beyond *Apophysomyces variabilis*, infections due to *R. microsporus* and *R. homothallicus* are on the rise in India [5,142]. *R. homothallicus* was isolated from patients with ROCM type, pulmonary and cutaneous form of disease [5,143,144]. A single case of fatal pulmonary infection due to *R. homothallicus* was reported from France [145]. Infections due to uncommon *Mucorales* such as *Cokeromyces recurvatus*, *Syncephalastrum* species, and *Saksenaea* species are rarely reported [4,139]. *Mucor irregularis* infection was reported from China and India [146,147]. Infection due to *Mucor velutinosus* and *Mucor ellipsoideus* have been reported from the USA [148]. A case of rhino-orbito-cerebral mucormycosis due to *Thamnostylum lucknowense* was reported from India [149]. *Mucorales* are thermo-tolerant fungi, ubiquitous in the nature, and are widely found in organic decaying materials [2]. A detail ecological study in India showed an abundant presence of diverse *Mucorales* species in soil [150]. The so called rare mucormycete agents such as *Apophysomyces variabilis* and *Rhizopus homothallicus* are found in India soils. 

## 7. Conclusions

The exact burden of mucormycosis is not known, as it is not a reportable disease and rare in developed countries. The disease is common in developing countries, but the laboratory facility is sub-optimal in this region. The estimate of mucormycosis has been accessed from large series, though majority studies did not have any denominator. Few population-based studies estimated the incidence of mucormycosis in the western world, but the same does not represent the picture in developing countries, as the risk groups and interventions are different between these two worlds. In developing countries, the incidence of diabetes mellitus is alarmingly high, which may lead to a rise in the incidence of mucormycosis. In developed countries, the rise in incidence of mucormycosis is linked to intense immunosuppression in haematological malignancies and transplant recipients. A change in epidemiology of mucormycosis has been noted in recent years with emergence of new risk factors and causative agents. Post-tuberculosis, chronic renal failure and stay in intensive care unit are new risk factors for the disease especially in developing countries. *Rhizopus homothallicus, Thamnostylum lucknowense*, and *Mucor irregularis* are the new emerging species. It warrants population-based studies on specific risk population (e.g., diabetes mellitus, transplant recipients) to understand global epidemiology of the disease.

## Figures and Tables

**Table 1:** The estimated burden of mucormycosis in different countries.

Country	Total Population (in millions)	Total Estimated Fungal Burden	Mucormycosis		Invasive Aspergillosis	
			Total Burden	Rate/100K	Total Burden	Rate/100K
Algeria [25]	40.4	568,942	79	0.2	2865	7.1
Argentina [26]	43.8	881,023	75	0.17	2536	5.8
Australia [27]	23.57	693,708	21	0.06	560	3–29%
Belgium [13,28]	11.1	233,000	31	0.58	675	6.08
Brazil [29,30]	194.0	3,800,000	243	0.2	8664	4.47
Cameroon [31]	24.2	1,126,332	5	0.2	1175	5.3
Canada [32,33]	35.5	652,932	43	0.12	566	1.59
Chile [34]	17.5	325,036	35	0.2	296	1.7
Colombia [35]	49.3	760,808	99	0.2	2820	5.7
Czech Republic [36]	10.5	176,073	22	0.2	297	2.8
Denmark [37]	5.6	894,430	1	0.02	294	5.3
Dominican Republic [38]	10.9	2,293,681	20	0.2	61	0.8
France [39]	65.8	968,143	79	0.12	1185	1.8
Greece [40]	10.8	194,067	7	0.06	1125	10.4
India [24]	1300.0	NA	171,504	14	NA	NA
Ireland [41]	6.4	117,384	13	0.2	445	7
Japan [42]	127.0	2,370,314	254	0.2	1308	1
Jordan [43]	6.3	119,153	1	0.02	84	1.34
Kazakhstan [44]	17.7	300,824	16	0.09	511	2.8
Kenya [45]	43.6	3,186,766	80	0.2	239	0.6
Korea [46]	48.0	985,079	68	0.14	2150	4.48
Malawi [47]	17.7	1,338,523	30	0.2	1186	6.7
Mexico [48]	112.3	2,749,159	134	0.12	4510	4
Nigeria [49]	155	17,983,517	300	0.2	928	0.6
Norway [50]	5.2	839,087	7	0.1	278	5.3
Pakistan [51]	184.5	3,280,554	25,830	14	10,949	5.9
Philippines [52]	98.4	1,852,137	20	0.02	3085	3
Portugal [53]	10.6	1,695,514	10	9.5	240	2.3
Qatar [54]	1.9	33,448	23	1.23	11	0.6
Romania [55]	19.7	436,230	7	0.04	1524	7.7
Russia [56]	142.9	3,082,907	232	0.16	3238	2.27
Serbia [57]	7.1	156,825	23	0.33	619	8.8
Spain [58]	47.0	8,144,605	20	0.04	1293	2.75
Thailand [59]	65.1	1,254,562	130	0.2	941	1.4
Ukraine [60]	45.5	999,152	90	0.1975	1233	2.7067
United Kingdom [61]	63.18	241,525–662,987	57	0.09	2901–2912	4.59–4.61
USA [62]	NA	NA	36	0.3	301	2.4
Republic of Uzbekistan [63]	30.7	536,978	27	0.08	1521	4.8

NA: data not available.
